# Transpedicular hydroxyapatite grafting with indirect reduction for thoracolumbar burst fractures with neurological deficit: A prospective study

**DOI:** 10.4103/0019-5413.37002

**Published:** 2007

**Authors:** Tomoaki Toyone, Tomoyuki Ozawa, Yuichi Wada, Koya Kamikawa, Atsuya Watanabe, Takeshi Yamashita, Keisuke Matsuki, Ryutaro Shiboi, Nobuhiro Matsumoto, Shunsuke Ochiai, Tadashi Tanaka

**Affiliations:** Department of Orthopedic Surgery, Teikyo University Chiba Medical Center, 3426-3 Anesaki, Ichihara-city, Chiba 299-0111, Japan; * Division of Orthopedic Surgery, Kimitsu Chuo Hospital, 1010 Sakurai, Kisarazu-city, Chiba 292-8535, Japan

**Keywords:** Pedicle screw fixation, thoracolumbar burst fracture, transpedicular hydroxyapatite grafting

## Abstract

**Background:**

The major problem after posterior correction and instrumentation in the treatment of thoracolumbar burst fractures is failure to support the anterior spinal column leading to loss of correction of kyphosis and hardware breakage. We conducted a prospective consecutive series to evaluate the outcome of the management of acute thoracolumbar burst fractures by transpedicular hydroxyapatite (HA) grafting following indirect reduction and pedicle screw fixation.

**Materials and Methods:**

Eighteen consecutive patients who had thoracolumbar burst fractures and associated incomplete neurological deficit were operatively treated within four days of admission. Following indirect reduction and pedicle screw fixation, transpedicular intracorporeal HA grafting to the fractured vertebrae was performed. Mean operative time was 125 min and mean blood loss was 150 ml. Their implants were removed within one year and were prospectively followed for at least two years.

**Results:**

The neurological function of all 18 patients improved by at least one ASIA grade, with nine (50%) patients demonstrating complete neurological recovery. Sagittal alignment was improved from a mean preoperative kyphosis of 17°to −2°(lordosis) by operation, but was found to have slightly deteriorated to 1° at final followup observation. The CT images demonstrated a mean spinal canal narrowing preoperatively, immediate postoperative and at final followup of 60%, 22% and 11%, respectively. There were no instances of hardware failure. No patient reported severe pain or needed daily dosages of analgesics at the final followup. The two-year postoperative MRI demonstrated an increase of one grade in disc degeneration (n = 17) at the disc above and in 11 patients below the fractured vertebra. At the final followup, flexion-extension radiographs revealed that a median range of motion was 4, 6 and 34 degrees at the cranial segment of the fractured vertebra, caudal segment and L1-S1, respectively. Bone formation by osteoconduction in HA granules was unclear, but final radiographs showed healed fractures.

**Conclusions:**

Posterior indirect reduction, transpedicular HA grafting and pedicle screw fixation could prevent the development of kyphosis and should lead to reliable neurological improvement in patients with incomplete neurological deficit. This technique does not require fusion to a segment, thereby preserves thoracolumbar motion.

Posterior instrumentation in the treatment of thoracolumbar burst fractures have allowed a decrease in the number of fused segments and indirect reduction technique has enabled greater canal clearance and correction of kyphosis.^1^,^2^ Despite these advantages, failure to support the anterior spinal column leads to eventual loss of correction and high rate of failure of posterior instrumentation. Cancellous bone-grafting to the fractured body by the transpedicular approach had been thought to provide some additional support to the anterior column. However, many researchers have reported that it could not decrease the loss of correction.^3^–^5^ We wondered why cancellous bone-grafting to the fractured body fails to support the anterior column. We speculated that cancellous bone would be absorbed before bone union is accomplished, but hydroxyapatite (HA) ceramics would not. We have reported a prospective clinical cohort study of patients with thoracolumbar burst fractures who were all treated with short segment posterior instrumentation supplemented with transpedicular HA augmentation of the fracture in the vertebral body.^6^ The neurological function of all 15 patients improved by at least one ASIA grade, with nine (60%) patients demonstrating complete neurological recovery. The mean loss of correction of kyphosis was 2 degrees and there were no instances of hardware failure. We hereby present the results of the additional cases in which we used new HA ceramics.

## MATERIALS AND METHODS

The inclusion criteria for this study included all of the following: (a) a single thoracic and lumbar burst fracture Type A3 according to a comprehensive classification of thoracic and lumbar injuries,^7^ demonstrated on both radiographs and computed tomography (CT) scan; (b) spinal cord or cauda equina injuries; (c) admission to the hospital within 24 h of injury; (d) possible to undergo surgery between 24-96 h after injury; (e) possible to have implants removed one year later. Patients with multiple life-threatening injuries, previous neurological injuries and pathologic lesions were excluded. From 2000 through 2005, 18 patients met the criteria. There were seven women and 11 men, ranging from 14 to 59 years of age (mean age: 38 years, standard deviation: 15) at the time of surgery [Table 1].

**Table 1:** Patients' demographics

Case	Age	Sex	Level	Spinal canal narrowing (%)			Vertebral kyphosis (degrees)		
					
				Peroperative	Postoperative	Final followup	Peroperative	Postoperative	Final followup
1	35	m	L3	67	33	11	−3	−14	−12
2	17	m	L3	67	22	11	3	−16	−15
3	24	f	L1	67	17	8	21	−1	1
4	46	m	L1	56	22	11	12	−2	−1
5	14	f	L2	58	17	8	25	1	3
6	50	m	L1	56	22	11	9	−6	−3
7	59	m	L1	67	22	11	20	9	11
8	31	f	T12	50	17	8	27	5	6
9	39	m	T12	60	20	10	21	4	6
10	35	m	L2	64	27	18	11	−4	−2
11	57	f	T12	56	22	11	20	4	1
12	53	m	L2	64	27	18	18	−4	−3
13	52	m	L1	60	20	10	22	−3	−2
14	21	m	T12	64	27	18	23	2	3
15	31	f	T12	60	20	10	20	−3	2
16	52	f	T12	56	22	0	21	0	5
17	22	f	L1	56	22	18	18	−1	3
18	54	m	L2	60	20	10	22	2	6

**Figure 1:**
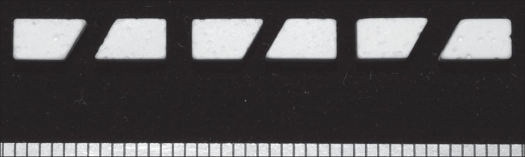
Hydroxyapatite blocks

HA sticks6 and HA blocks (Pentax, Tokyo, Japan, Figure 1) were used in 15 patients (case1-15) and three patients (Cases 16-18), respectively

Neurological assessment was made for each patient using a rating system based on that of the American Spine Injury Association (ASIA) impairment scale. Details of this five-level grading system are as follows: A) Complete: No motor or sensory function is preserved in the sacral segments S4-S5. B) Incomplete: Sensory but not motor function is preserved below the neurological level and extends through the sacral segments S4-S5. C) Incomplete: Motor function is preserved below the neurological level and majority of key muscles below the neurological level have a muscle grade less than 3. D) Incomplete: Motor function is preserved below the neurological level and the majority of key muscles below the neurological level have a muscle grade more than or equal to 3. E) Normal: Motor and sensory function is normal. Neurological level was noted as the most caudal segment with normal function.

Radiographic assessment was performed using anteroposterior and lateral roentgenograms and CT scan of the area of injury. The vertebral kyphosis was measured from the superior end-plate of the intact vertebra cephalad to the fracture to the inferior end-plate of the vertebra caudal to the fracture. Canal stenosis was determined on CT by directly measuring the mid sagittal anteroposterior canal dimension at the injured level and was reported in millimeters. This figure was then compared with the average of similar dimensions measured at the levels above and below the injury level. The result of this comparison was reported as per cent anteroposterior canal compromise at the injury area. The extent of intervertebral disc degeneration was graded on midsagittal T2-weighted MR images according to the criteria of Borenstein *et al*.^8^ as follows: Normal (Score 0), Mild (Score 1); slight dehydration of the disc on T2-weighted images, Moderate (Score 2); disc dehydration and mild loss of disc height and Severe (Score 3); total disc dehydration with nearly complete loss of disc height. Each disc level, above and below the fractured vertebra, was graded.

All surgical procedures were performed by first author (TT) under controlled general anesthesia with endotracheal intubation. Patients were placed in a prone position on a radiolucent operating table. Using a standard posterior midline approach, Schanz screws (AO Universal Spine System) were placed down the pedicles of the vertebrae below and above the fracture. Posterior wall decompression was achieved by indirect reduction via ligamentotaxis by the following technique.^9^ The lordosing maneuver was performed first followed by segmental traction. Prior to reduction with the Schanz screws, two half-rings (or rod holders) were placed on each of the 6-mm rods, at a distance of 5 mm from the clamp of the Schanz screw, in order to protect the posterior wall of the vertebral body from compression. Then we corrected the kyphosis by manually approximating the dorsal ends of the Schanz screws. Further, we applied distraction by using spreader forceps checking the procedure with an image intensifier. We made sure not to apply too much distraction to the adjacent discs in order to avoid loss of correction.

Following indirect reduction and fixation, a 6-mm diameter hole was made into the pedicle of the fractured vertebra initially with the pedicle probe. The position was controlled by an image intensifier, which was then enlarged with the use of an access cannula with a trocar. Once the cannula reached the center of the vertebral body, under continuous fluoroscopic monitoring, HA ceramics were pushed anteriorly into the defect region. The HA sticks^6^ and HA blocks (Pentax, Tokyo, Japan, [Figure 1]) were used in 15 and three patients, respectively. The HA insertion was considered complete when it reached the posterior third of the vertebral body. When the volume delivered via a single pedicle was thought to be insufficient in cases where HA did not reach the midline of the vertebral body on AP projection, we repeated this technique on the opposite pedicle. No autologus iliac bone graft was used. We did not manipulate any of the vertebral body fracture fragments or elevate the fractured vertebral body endplate.

Patients were allowed to walk while wearing a three-point fixation brace on the following day after surgery. The vigorous work and activity were restricted up to 12 weeks postoperatively. All patients were postoperatively followed clinically and radiographically at one week, one month, one year around the time of rod removal and at every one-year interval thereafter. As for CT scan, all patients had it just after surgery, one year later and two years later. The mean followup period was 44 months (range, 24-60 months).

## RESULTS

The distribution of injury levels was as follows: T12 (n=6); L1 (n=6); L2 (n=4); L3 (n=2). According to the Denis classification, 10 patients sustained Type A (both endplate fracture) and eight Type B (superior endplate fracture).^10^ The radiological examinations of all the patients at the time of injury did not demonstrate obvious findings of posterior ligamentous disruption and elongation. Mean operative time was 125 min (range, 75-155 min) and mean blood loss was 150 ml (range, 50-227 ml). The volume of HA required ranged from 6 to 12 g.

**Figure 2:**
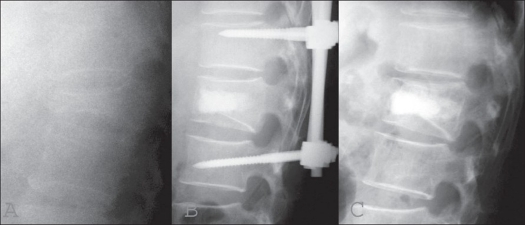
Lateral X-ray of thoracolumar spine of a 59-year-old man had a burst fracture of the first lumbar vertebra, with a neurological deficit, of ASIA Grade C on admission (Case 7). Lateral X-ray (A) A 59 years old man shows burst fracture of L1 vertebra. The patient was ASIA grade C on admission. The patient has vertebral kyphosis (T12-L2) 20 degrees. Immediate Post-operative lateral x-ray of dorso lumbar spine (B) shows post instrumentation anterior HA cylindrical stick and vertebral kyphosis corrected to 90. Standing lateral roentgenogram (C) taken two years after the operation showing re-establishment of sagittal alignment. Vertebral kyphosis had slightly deteriorated to 11 degrees. This patient had recovery of neurological loss of ASIA Grade from C to D

**Figure 3:**
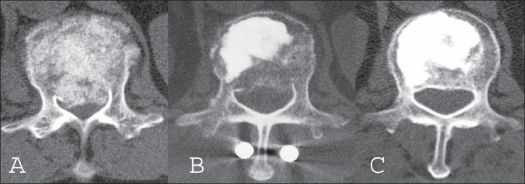
Axial CT scan (A) image shows posterior cortical bone retropulsion. and spinal canal narrowing of 67%. Postoperative Axial CT scan (B) following surgery demonstrating adequate HA graft placement and reduction of the retropulsed bony fragment. Axial CT scan (C) of 2 years after surgery demonstrating further improvement of the spinal canal narrowing and radiographic healing of the vertebral body

Radiographic assessment of the lateral plane demonstrated that mean vertebral kyphosis was 17 degrees (range, from −3 to 27 degrees) before surgery, was corrected to −2 degrees (lordosis) (range, from −16 to 9 degrees) postoperatively, but was found to have slightly deteriorated to 1 degree (range, from −15 to 11 degrees) at final followup [Table 1]. The CT images demonstrated a mean spinal canal narrowing of 60% (range, 50 to 67%) before surgery, 22% (range, 17 to 33%) postoperatively and 11% (range, 8 to 18%) at final followup showing further improvement [Figure 2–3]. At the time of implant removal, there was no failure of posterior instrumentation including breakage, bending or toggling of the pedicle screw.

**Figure 4:**
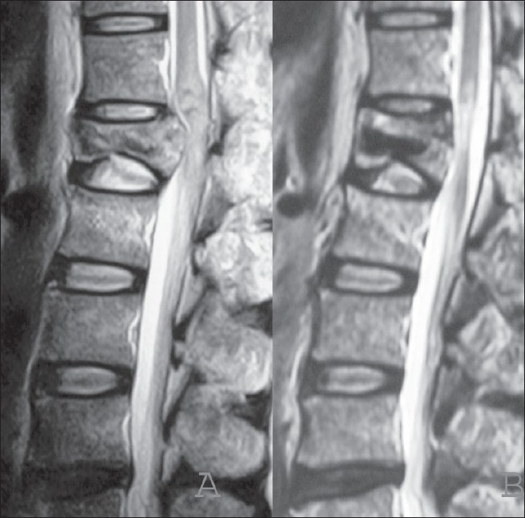
(A) Preoperative T2WI mid sagittal MR imaging showing severe compression of the dural sac by bone retropulsion. Three-year postoperative sagittal MR imaging (B) demonstrating decompression of the spinal canal and signal intensity regression of the disc above the fractured vertebra

Two-year postoperative MRI demonstrated an increase disc degeneration of one grade at the disc above and below the fractured vertebra in all eight subjects who had both endplate fractures and at the disc above the fractured vertebra in all 10 subjects who had superior endplate fracture [Figure 4]. At the final followup, flexion-extension radiographs revealed that the mean range of motion was 3, 6 and 35 degrees at the cranial segment of the fractured vertebra, the caudal segment and L1-S1, respectively. No subject achieved spontaneous anterior interbody fusion. Bone formation by osteoconduction in HA ceramics was unclear, but final radiographs showed healed fractures.

Based on the ASIA neurological grading system, one patient was Grade B, 10 were Grade C and seven were Grade D before surgery. All 18 patients demonstrated at least one ASIA grade neurological improvement on final followup observation. Two patients who were ASIA Grade C and seven patients who were ASIA Grade D, demonstrated complete neurological recovery. One patient who was ASIA Grade B and eight patients who were ASIA Grade C improved to ASIA Grade D.

Twelve patients did not report back pain on followup. Five reported mild pain and one reported moderate pain. None reported severe pain or needed daily dosage of analgesics.

## DISCUSSION

In the treatment of thoracolumbar burst fractures, the neural elements and the stability and alignment of the vertebral column, are most important considerations. Posterior instrumentation have allowed a decrease in the number of fused segments and indirect reduction technique has enabled greater correction of kyphosis. Despite these advantages, there are two major problems. The first is that ligamentotaxis is not always successful and produces incomplete decompression of the spinal canal. Although surgery to remove retropulsed bone fragments directly from the canal in burst fractures to decompress the spinal canal has been suggested,^11^–^13^ most studies show little correlation between the degree of narrowing of the canal and the neurological injury sustained.^10^,^14^–^16^ There is, similarly, a poor relationship between the decompression achieved and the neurological recovery ensured.^7^,^17^,^18^ Neurological damage occurs at the moment of injury.^16^ Boerger *et al*. retrieved 275 publications on the management of burst fractures and concluded that surgery may occasionally be indicated for structural reasons, not for neurological improvement.^19^ Further, some reports have shown partial or total resorption of retropulsed bone fragments and spontaneous remodeling of the spinal canal, as shown in this study.^20^–^23^

The second major problem is the failure to support the anterior spinal column after posterior correction and instrumentation. McLain *et al*. reported that 10 of the 19 patients without fusion had some form of failure of the fixation during their early period of healing.^6^ Benson *et al*. reported that the initial correction of the traumatic sagittal plane deformity was not maintained and deteriorated over time despite a solid posterolateral fusion.^24^ Berlemann *et al*. reported on 20 patients and stated that indirect reduction and posterior instrumentation could initially reduce the segmental kyphosis completely, but posterolateral fusion alone did not prevent disc space collapse and eventually, led to loss of correction.^25^ Although longterm results were favorable, these authors recommend anterior interbody fusion. Knop *et al*. reported that the addition of transpedicular cancellous bone grafting did not decrease the loss of correction in 33 patients.^4^ Walchli *et al*. reported 15 patients with or without intracorporal transpedicular bone grafting and noted that neither posterolateral fusion nor posterolateral fusion with transpedicular bone grafting could prevent loss of angular corrections.^5^ Alanay *et al*. randomized 21 patients into transpedicular grafting (n=11) and non-transpedicular grafting (n=10) and prospectively followed them, but there was no difference between groups regarding canal clearance and sagittal deformity.^6^

The mean loss of correction of kyphosis ranging from 3 to 12 degrees was reported in the clinical results of short-segment pedicle-screw fixation.^3^,^18^,^24^ The mean loss of correction in our series was 3 degrees, which was similar to those of the series of anterior decompression and stabilization with the Kaneda device (1°)^13^ and Z-plate (2°).^26^ It was reported that the rate of failure of posterior instrumentation including breakage, bending or loosening of the pedicle screw ranged from 9 to 54%.^3^,^18^,^24^,^27^ No instrumentation failure was observed in the present study, which was thought to be due to adequate support of the anterior column provided by transpedicular HA grafting.

Mermelstein *et al*. biomechanically studied the reinforcement of thoracolumbar burst fractures with HA cement.^28^ They used a cadaveric burst fracture model which was stabilized using short segment pedicle screw instrumentation and reinforced it with HA cement through a transpedicular approach. They reported that transpedicular vertebral body reconstruction with HA cement reduced pedicle screw-bending moments and significantly increase initial stiffness in the flexion-extension plane. Li *et al*. used titanium block designed for transpedicle body reconstruction and reported that posterior body reconstruction with a transpedicle body augmenter can maintain kyphosis correction and vertebral restoration, prevent implant failure and lead to better clinical results.^29^ Cho *et al*. treated thoracolumbar burst fractures with polymethyl methacrylate (PMMA) vertebroplasty and short segment pedicle screw fixation and reported that PMMA vertebroplasty offers immediate spinal stability in patients with thoracolumbar burst fractures, decreases the instrument failure rate and provides better postoperative pain control than without vertebroplasty.^30^

Narrowing of the disc space has commonly been observed after thoracolumbar burst fractures and has been associated with recurrent kyphosis after posterior reduction and fixation.^31^ Oner *et al*. reported that the signal intensity of the discs was preserved and most changes were related to morphological alterations in the disc space after fractures of the endplate.^32^ We considered longterm superior adjacent segment stability since that is the facet that can be injured with this flexion-compression injury. Although we observed progression of the disc degeneration adjacent to fractures, there was loss of motion at these segments, which may have been due to fixing the facet joints for one year. No patient reported severe back pain or needed daily dosages of analgesics.

The final clinical and radiological results of this study were gratifying and compare favorably with previous outcomes of the posterior approach. Comparing the results of this study and those of the recent series of anterior decompression and stabilization with the Z-plate,^26^ the former seems to be inferior to the later in postoperative canal compromise, but superior to the later in kyphotic deformity correction, surgical time and blood loss.

Several important points of this procedure should be brought up at the end of this paper. If the reverse fragment is demonstrated on CT scan, it should be associated with the rupture of the posterior longitudinal ligament and such a case should not be indicated for this procedure. Although posterior wall decompression should be achieved by way of indirect reduction via ligamentotaxis, not too much distraction should be applied to the adjacent discs in order to avoid loss of correction.
